# Strengthening face centered cubic crystals by annealing induced nano-twins

**DOI:** 10.1038/s41598-017-17848-3

**Published:** 2017-12-13

**Authors:** Barna Roy, Jayanta Das

**Affiliations:** Department of Metallurgical and Materials Engineering, Indian Institute of Technology Kharagpur, West Bengal, 721302 India

## Abstract

Usually, cold working strengthen metals and alloys by introducing large population of dislocations, whereas annealing of cold worked metal recovers the structure, annihilates dislocations, forms new strain-free grains, and results loss of strength. Here, we report annealing-hardening at temperature well below stress relieving and recrystallization temperatures in contrast to the typical behavior. A large amount of structural defects, such as dislocations, grain boundaries, twins, and stacking faults, have been introduced in nanostructured α-brass by cryorolling. The interaction and rearrangement of these defects upon annealing at 165–200 °C have been monitored at an interval of 1 minute. Large increase of the yield strength up to 578 MPa has been achieved in annealed specimens, which is 23% higher than that of as-cryorolled, and 425% higher than that of as-cast brass due to the evolution of nano-twins. Our approach shows a new avenue on strengthening fcc crystals by incorporating annealing induced nano-twins.

## Introduction

Extensive studies have been performed to reveal the effect of deformation twinning on strengthening of fcc crystals^1–8^. Furthermore, $\frac{a}{2}[10\bar{1}]$ perfect dislocations are dissociated into $\frac{a}{6}[2\bar{1}\bar{1}]$ and $\frac{a}{6}[11\bar{2}]$ Shockley partials by forming wider stacking fault (SF) ribbons, which restrict the cross-slip and the climb of dislocations in low stacking fault energy (SFE) metal/alloys^9^. Therefore, twinning is the preferred mode of deformation in such low SFE material and the recovery of dislocations by either cross-slip or climb becomes difficult when they encounter barriers^7,9,10^. The strength of the twinned crystals with high dislocation density have been correlated with dislocation mean free path according to the Hall-Petch-type equation^7,11,12^:

1 $${\sigma }_{y}={\sigma }_{o}+k{(\frac{1}{{d}_{twin}}+\frac{1}{D})}^{\frac{1}{2}}+\alpha Gb\sqrt{{\rho }_{d}}$$

where, *σ*_*y*_ is the flow stress, *d*_*twin*_ is the twin spacing, *D* is the crystallite size, *G* is the shear modulus, *b* is the value of Burger’s vector, *ρ*_*d*_ is dislocation density, *σ*_0_, *k*, and *α* are constants. However, annealing of such microstructure decreases the strength due to detwinning, evolution of low energy sub-structure, such as dislocation polygonization, annihilation of dislocations at high angle grain boundaries followed by recrystallization^10,13–15^.

So far, enhancement of strength or hardness in fcc crystals upon annealing, has been reported due to (i) the structural and crystallographic changes of the high density shear bands those evolved during cold rolling, which increase the flow stress by 3–10%^16^, (ii) thermal agitation causing the dislocation nucleation and their rearrangements in the cell and subgrain walls of a machined chip leading to 2–5% increase of the hardness^17^, and (iii) the clustering of solute atoms at SF, i.e., the ‘Suzuki effect’, which reduces the lattice parameter resulting an increase of microhardness by 9% of a cryorolled bar^18^.

α-brass (Cu-30 wt.% Zn) with fcc crystal structure possesses low SFE of 14 mJ/m^2^, which plays a key role on the deformation twinning^19–23^. Zn atoms agglomerate in the fcc-Cu lattice and change the stacking sequence by introducing an extra Zn layer in the parent lattice either above or below the fault and reduce the SFE^24^. Moreover, Zn helps in retaining the dislocation density during deformation, restricts cross-slip, and prevents dynamic recovery^10^. In conventional recrystallization annealing, both the structural defects and their density are reduced, whereas the internal microstress and residual stress are reduced during stress relieving annealing^25^. It will be interesting to study what will happen when the fcc crystals with high twin density (>10^6^/m) and high dislocation density (>10^14^/m^2^) are annealed at low temperatures (0.18T_m_–0.22T_m_).

In this work, we show a large increase of the flow stress of cryorolled α-brass upon annealing at temperatures of 165 °C and 200 °C, which are well below the stress relieving temperature. Interestingly, the high density of dislocations and nano-twins, those evolved upon cryorolling, were not annihilated completely during annealing. Whereas, the evolution of annealing induced nano-twins along with the prior dislocations and twins cause the anomalous hardening phenomena.

## Results

### Sample processing and measurement of yield strength

We have cryorolled (CR) α-brass up to a true strain of 0.4 (CR04) along thickness, which was further annealed at 165 °C and 200 °C for different soaking time. The experimental details are described in the methods and the choice of annealing temperatures is illustrated in Supplementary Fig. S1, which is well below recrystallization and stress relieving temperature.

**Figure 1:**
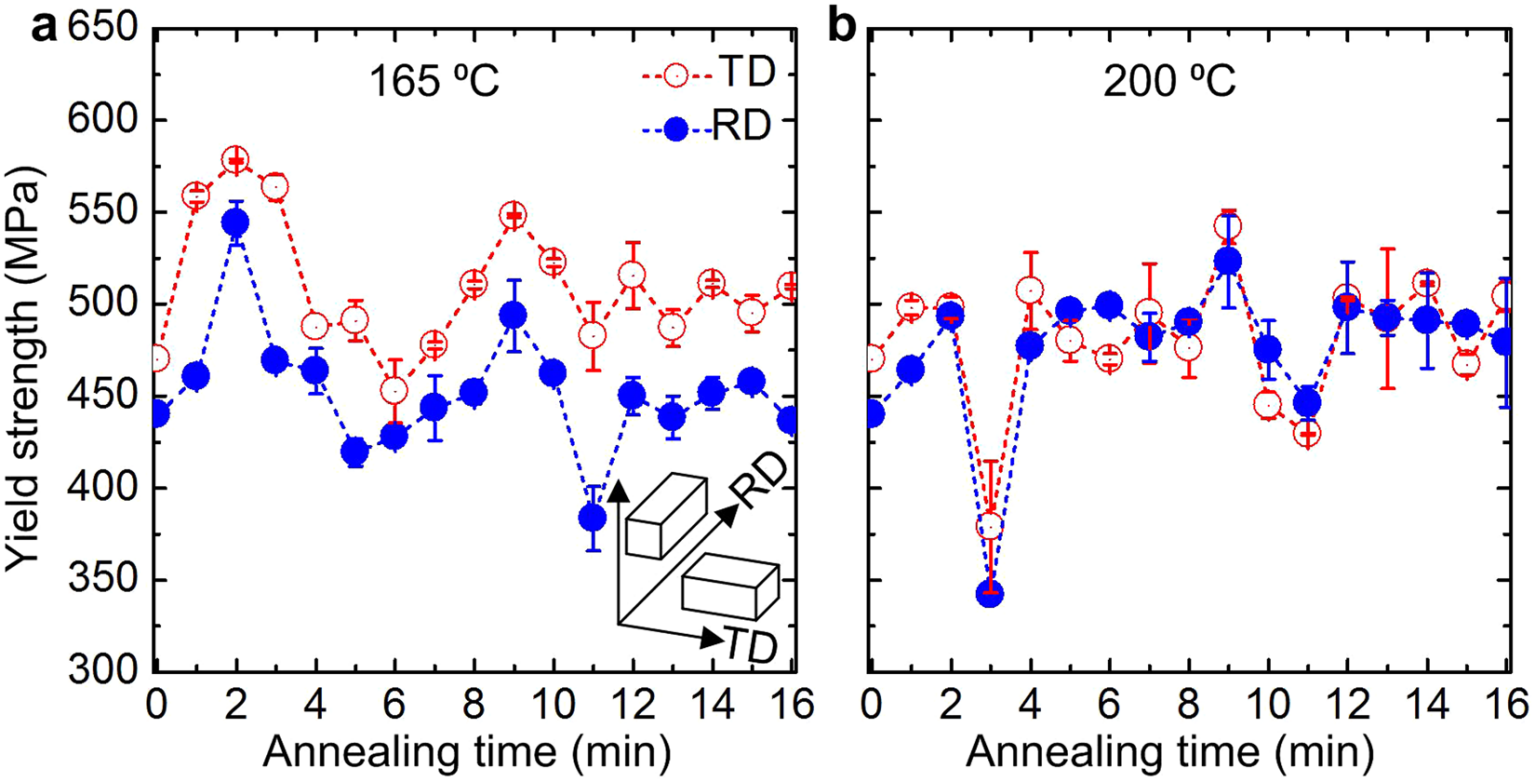
Anomalous increment of flow stress as a function of annealing time and temperature. (**a**,**b**) The effect of time on the variation of flow stress upon annealing at 165 °C (**a**) and 200 °C (**b**) along RD and TD. The inset of (**a**) shows the schematic of RD and TD from where the samples are collected for flow stress measurement.

We have measured yield strength of bulk specimens at room temperature under compression. The specimens were cut from the CR04 and differently annealed bars along rolling direction (RD) and transverse direction (TD) to reveal the effect of annealing time and temperature on the yield strength. The strength of CR04 is 440 MPa and 470 MPa along RD and TD, respectively, which is 4 times higher than that of as received solution treated (110 MPa) alloy^18^. Figure 1a,b, shows the variation of yield stress with annealing time along both the directions at 165 °C and 200 °C, respectively. Annealing at 165 °C increases the strength gradually with time and the strength reaches to a peak value of 544–578 MPa at 2 min. Thereafter, the strength decreases with time and reduces down to 423–450 MPa in between 5–6 min. Interestingly, further annealing increases the strength to 493–548 MPa at 9 min, which again reduces down to 383–482 MPa at 11 min. At 12 min of annealing, the yield stress reaches to 447–510 MPa and a negligible variation of the yield stress with time has been observed in between 12–16 min. On the other hand, annealing at 200 °C shows a little increase of strength (493–498 MPa) after 2 min compare to 165 °C. The fall of the yield stress (342–370 MPa) is also sharper for 3 min of annealing at 200 °C. Whereas, a remarkable increase of strength up to 523–542 MPa was observed for 9 min of annealing at 200 °C. Beyond 9 min, the variation of yield stress with time shows similar trend as of 165 °C. It should be noted that the strength values follow similar trend for both RD and TD for a given annealing time and are independent on sample orientation, as shown in Fig. 1a,b.

### Quantitative estimation of twin fault probability (*β*), dislocation density (*ρ*_*d*_), and crystallite size (*D*) in as-cryorolled and annealed specimens

**Figure 2:**
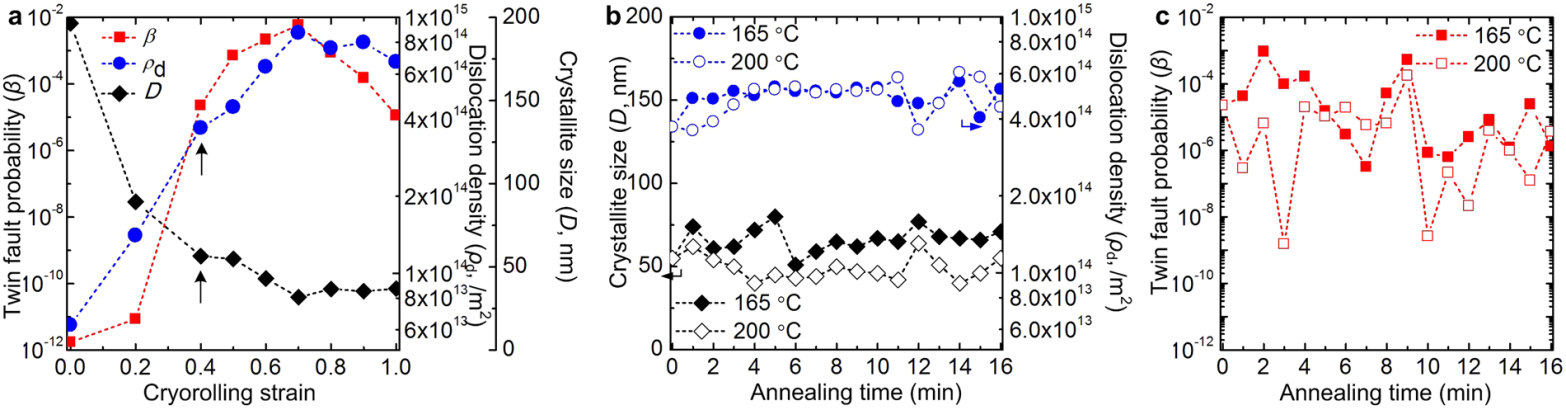
Evolution of various structural defects during cryrorolling and subsequent annealing. (**a**) Plot showing the variation of crystallite size (*D*), dislocation density (*ρ*_*d*_), and twin fault probability (*β*) during cryorolling. (**b**) The variation of *D* and *ρ*_*d*_ with annealing time at 165 °C and 200 °C. (**c**) The effect of annealing time on the evolution of *β* during annealing at 165 °C and 200 °C.

We have conducted x-ray peak broadening analysis to estimate the lattice parameter, *β*, *ρ*_*d*_, and *D* in the CR and annealed specimens, and their values are given in Supplementary Table S1. The lattice parameter of the CR and differently annealed specimen has been estimated to be 3.693 ± 0.001 Å, indicating that no phase transformation had occurred upon annealing. We have chosen CR04 specimen for annealing, as it contain 57 nm size crystallites along with high value of *ρ*_*d*_ (3.7 × 10^14^ /m^2^) and *β* (2.27 × 10^−5^). These *ρ*_*d*_ and *D* values are close to the peak values achieved in differently CR specimens, as shown in Fig. 2a. Figure 2b shows the changes in *D* and *ρ*_*d*_ with annealing time at both the temperatures. It has been clearly observed that the changes in *D* and *ρ*_*d*_ are negligible with time at both the temperature (see also Supplementary Table S1). Therefore, annealing at 165 °C and 200 °C up to 16 min neither initiate recovery nor cause recrystallization of the CR specimens. Whereas, the change in *β* with annealing time at 165 °C, is significant showing sharp peaks at 2 min and 9 min with a peak value of 9.3 × 10^−4^ and 5.3 × 10^−4^, respectively, as shown in Fig. 2c. These values are at least one order magnitude higher than that of CR04 (*β* = 2.3 × 10^−5^). On the other hand, the peak value *β* reaches to 1.8 × 10^−4^ after 9 min of annealing at 200 °C (empty bubble in Fig. 2c) (see also Supplementary Table S1). Moreover, the gradual decrease of *β* occurred immediately after 2 min and 9 min of annealing at both the temperatures indicating detwinning.

### Evolution of microstructure during cryorolling and annealing

**Figure 3:**
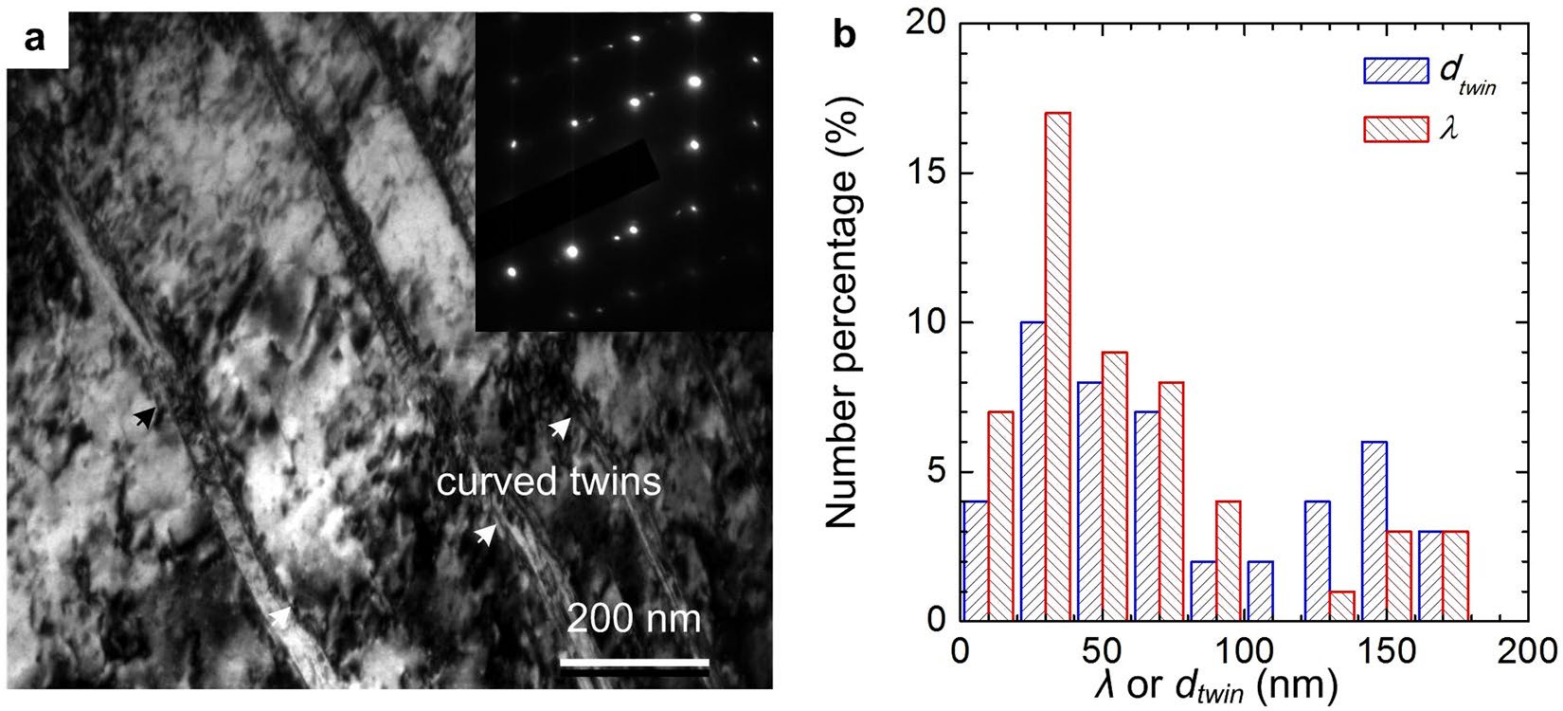
Microstructures of the as-cryorolled (CR04) specimens. (**a**) TEM (BF) micrograph of CR04 showing the presence of 50–60 nm thick deformation twins with curved boundaries. The inset shows the corresponding SAED pattern with twin diffraction spots. (**b**) Distribution of twin lamellae thickness (*λ*) and twin spacing (*d*_*twin*_) in CR04.

Transmission electron microscopic (TEM) image and the corresponding selected area electron diffraction (SAED) pattern of CR04 are shown in Fig. 3a. Detail investigation of the bright field (BF) micrograph and the double diffraction spots in the SAED pattern, as shown in Fig. 3a, reveals the presence of alternate lamellae of twin and matrix in the microstructure. The curved boundaries of the twins confirm that these are deformation twin. Recent investigations by Wang *et al*.^26^ and Ni *et al*.^27^ have shown that curved TBs are formed in fcc metals/alloys due to the accumulation of high density dislocations at TBs. Moreover, a high-density dislocation networks has been observed in the BF micrograph of CR04 (Fig. 3a). The twin lamellae (*λ*) and matrix lamellae thickness (*d*_*twin*_) have been measured from a large number of BF and the dark field (DF) micrographs. The distribution of *λ* and *d*_*twin*_ with average value of *λ* = 61 ± 7 nm and *d*_*twin*_ = 102 ± 11 nm in CR04 specimen are shown in Fig. 3b.

**Figure 4:**
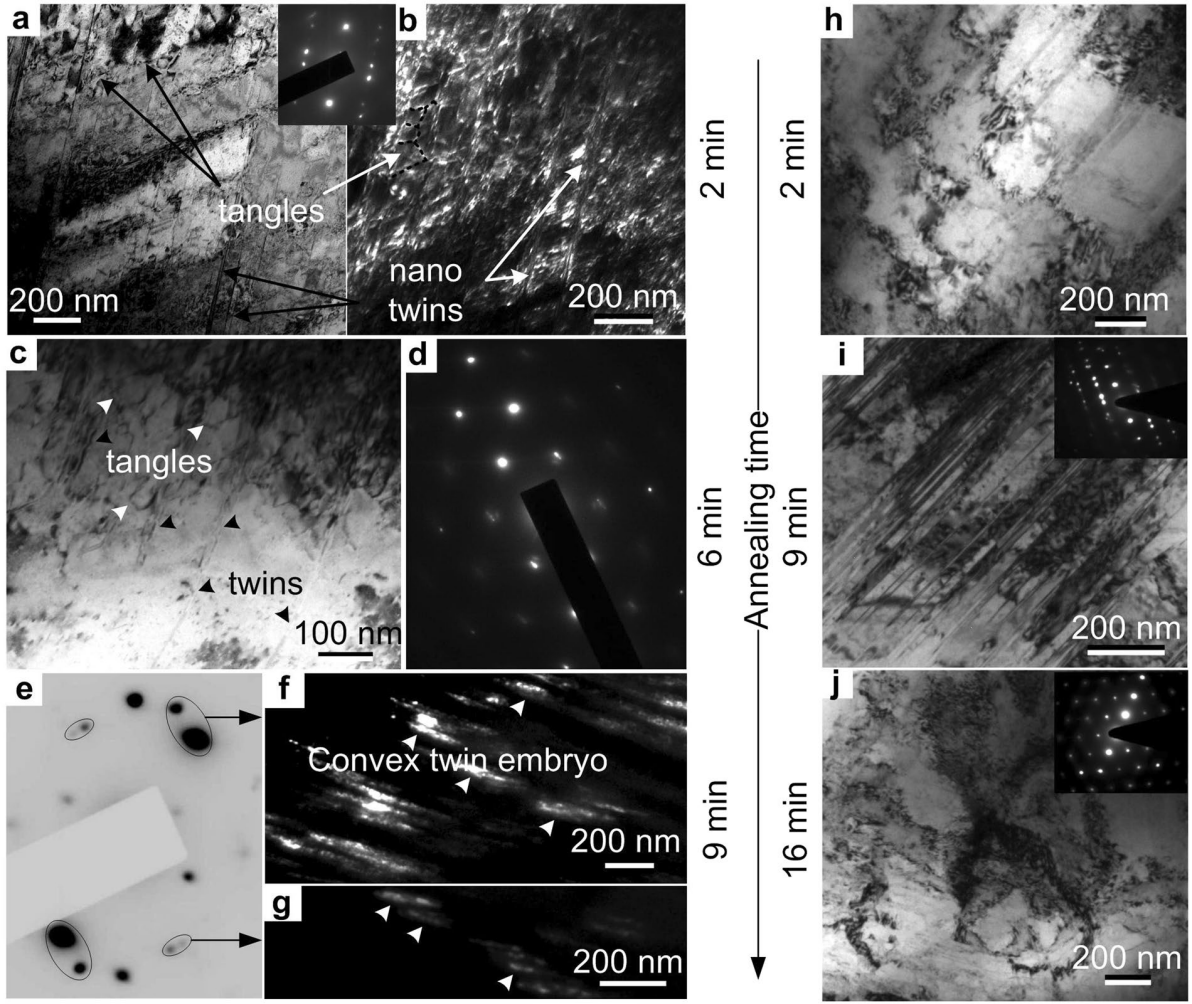
Evolution of microstructures upon annealing. (**a**,**b**) BF (**a**) and DF (**b**) TEM micrographs of the specimen annealed at 165 °C for 2 min showing the presence of 20–100 nm thick twins along with 50 nm size dislocation cells/tangles homogeneously distributed in the matrix. The inset shows the corresponding SAED pattern. (**c**,**d**) TEM BF micrograph (**c**) and corresponding SAED pattern (**d**) of the specimen annealed for 6 min at 165 °C showing the presence of very few twins in the microstructure. (**e**) SAED pattern obtained from the specimen annealed for 9 min at 165 °C. (**f,g**) DF micrographs of the corresponding spots in **(e)** showing the evolution of convex shape twin embryo upon annealing. (**h**) BF micrograph of the specimen annealed for 2 min at 200 °C showing the formation of 200–700 nm size dislocation tangles with very few deformation twins. (**i**) The microstructure of the 9 min annealed specimen at 200 °C showing the presence of abundant needle shape twins. The inset shows the corresponding SAED pattern with twin diffraction spots. (**j**) BF micrograph of the specimen annealed for 16 min at 200 °C showing the formation of 200–700 nm size dislocation tangles with negligible amount of twins. The inset shows the corresponding SAED pattern.

The TEM micrographs of the specimen annealed for 165 °C are shown through Fig. 4a–g. Annealing at 165 °C for 2 min introduces 20–100 nm thick twins in the microstructure, as shown in Fig. 4a,b. The BF and the DF micrographs of the same specimen also show the presence of ≤50 nm size dislocation cells/tangles, which are evenly distributed throughout the matrix. Dislocation networks have been often observed along with very few twins after 6 min of annealing at 165 °C, as shown in Fig. 4c, and the corresponding SAED pattern shown in Fig. 4d, respectively. Figure 4e–g depicts the SAED pattern and the corresponding DF micrographs of the specimen annealed for 9 min at 165 °C. Convex shape twin embryos were revealed to lie along different directions through out the microstructure, as marked by white arrows in Fig. 4e,g. Whereas, the BF TEM micrograph and the corresponding SAED pattern of the 16 min annealed specimen shows the presence of 200–500 nm size dislocation cells/tangles with negligible amount of twins (Supplementary Fig. S2).

The TEM BF images of the specimen annealed at 200 °C are shown in Fig. 4h–j. Figure 4h shows the BF micrograph of the specimen annealed for 2 min, which contain 200–700 nm size dislocation cells along with very few deformation twins. The micrograph of the specimen annealed for 9 min, as shown in Fig. 4i, reveal the presence of abundant needle shape twins with straight boundaries, which has also been confirmed by the twin diffraction spots in the SAED pattern, as shown in the inset of Fig. 4i. The straight twin boundaries indicate that these twins are annealing twins. Whereas, the micrograph of the specimens annealed for 16 min is almost similar to that of 2 min with 200–700 nm size dislocation cells and negligible amount of twins as shown in Fig. 4j.

### Correlation between twin density and Hall-Petch equation

**Figure 5:**
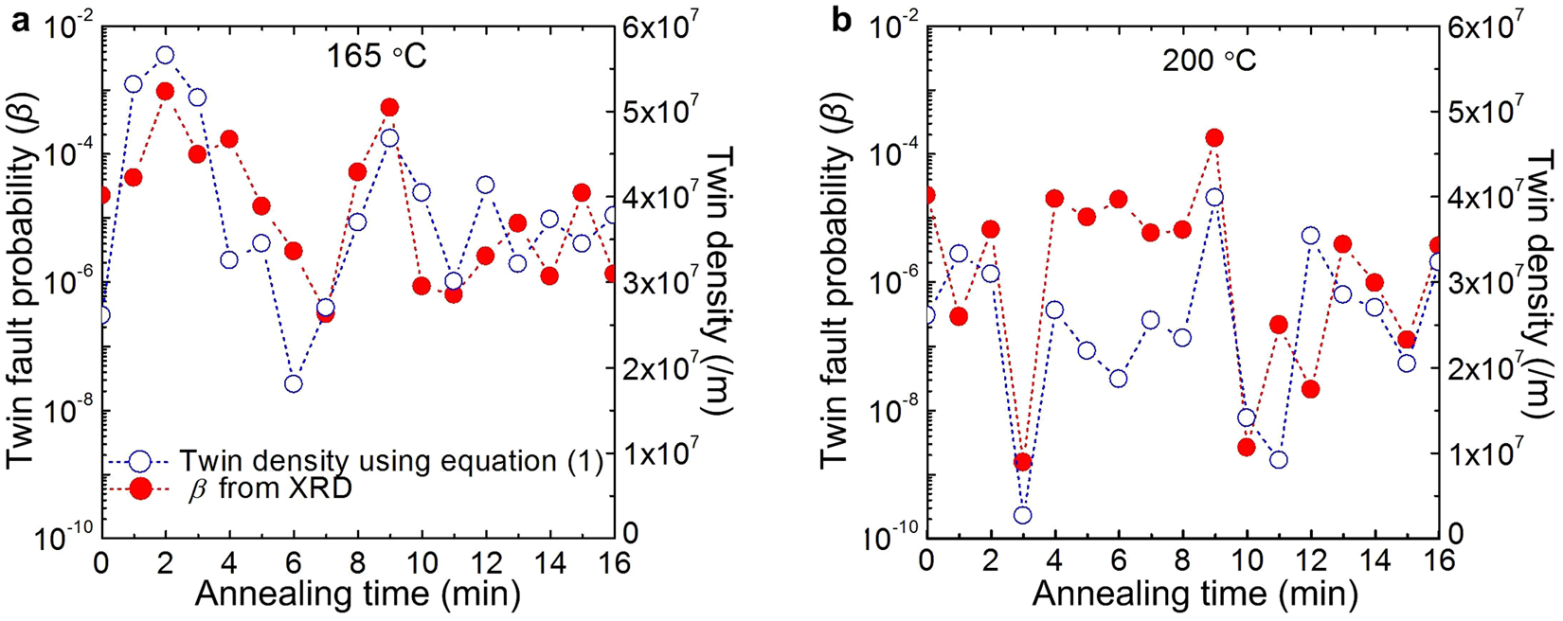
Comparison between twin fault probability (*β*) as estimated from the XRD peak broadening analysis, and twin density as estimated using equation (1) considering the dislocation density (***ρ***_***d***_) and crystallite size (*D*). (**a**,**b**) Plot showing the similar trend of *β* and twin density at 165 °C (**a**) and 200 °C (**b**) with annealing time. The anomalous hardening at 165 °C (2 min and 9 min) and 200 °C (9 min) are linked with the evolution of annealing induced nano-twins only.

We have calculated the twin density using equation (1) and have compared with that of as obtained from XRD studies and TEM analysis. In addition, we have considered the *ρ*_*d*_ and *D* values in equation (1) as estimated from XRD peak broadening analysis and *σ*_*y*_ is the compressive flow stress of the CR and differently annealed specimens. The values of *σ*_*o*_ and *k* have been taken as 81 MPa and 1.7 MPa√mm, respectively, from our earlier report^19^. The twin density has been estimated to vary in between 1 × 10^7^/m–6 × 10^7^/m, as shown in Fig. 5a,b for 165 °C and 200 °C, respectively. These values scale with the twin density in CR02 (8.7 × 10^6^/m) and CR10 (2.6 × 10^7^/m), as measured from TEM BF and DF micrographs of the differently CR specimens^19^. In addition, the variation of twin density in differently annealed specimens, as calculated using equation (1), follows similar trend to that of the *β*, as estimated from XRD peak broadening analysis for both the temperatures (Fig. 5).

## Discussion

The evolution of nano-size annealing twins without initiating the recovery upon low temperature annealing is the key to achieve anomalous hardening phenomena in nanostructured α-brass. In our previous study, we have estimated the value of *ρ*_*d*_ and the *β* of solution treated α-brass to be 6.3 × 10^13^/m^2^ and 1.9 × 10^−13^, respectively, which indicate the microstructure contain negligible amount of defects^21^. Our experimental results show that cryorolling introduces high density of dislocations (3.70 × 10^14^/m^2^) and deformation twins (*β* = 2.27 × 10^−5^, *d*_*twin*_ = 61 ± 7 nm) in the microstructure. Moreover, the plot between *ρ*_*d*_ and annealing time (Fig. 2b) at both the temperatures reveals that *ρ*_*d*_ remain constant with annealing time and the dislocations were not annihilated upon annealing. Whereas, the TEM micrograph (Fig. 4) shows that the dislocations were rearranged to form dislocation cells/tangles and the dislocation cell size became coarser as the duration of annealing increases. Therefore, annealing does not annihilate the high-density dislocations those evolved during cryorolling, but rearranges them to form dislocation cells/subcells with low energy configuration.

The peak value of *β* (9.28 × 10^−4^) at 2 min of annealing at 165 °C (Fig. 2c) along with the presence of abundant TBs in the TEM BF (Fig. 4a) and DF (Fig. 4b) micrographs confirm the nucleation of annealing twins in the microstructure. On the other hand, the reduction of *β* beyond 2 min of annealing is due to the initiation of the detwinning process, which has been confirmed by the presence of very few twins in the TEM BF micrograph (Fig. 4c) and weak twin diffraction spots in the SAED pattern (Fig. 4d) of the specimen annealed for 6 min. Moreover, the sharp increase in *β* for 9 min of annealing (Fig. 2c) along with the presence of convex shape twins in the TEM DF micrographs (Fig. 4f,g) confirms that these new twins have nucleated upon annealing. In case of annealing at 200 °C, besides a sharp peak at 9 min the *β* is almost equal or lower than that of as rolled specimen, as shown in Fig. 2c. The specimen annealed for 9 min shows the presence of abundant nano-twins with straight boundaries, indicating that new twins have nucleated during annealing for only 9 min at 200 °C and are responsible for the increase of *β* value. The presence of negligible amount of twins in the TEM micrograph and the corresponding SAED pattern of the specimens annealed for 2 min (Fig. 4h) and 16 min (Fig. 4j) reflects the lower activity of twinning at 200 °C. Therefore, annealing of CR α-brass below stress relieving temperature alters the microstructure by rearranging the dislocations cells/sub-cells along with the dissociation and nucleation of twins.

**Figure 6:**
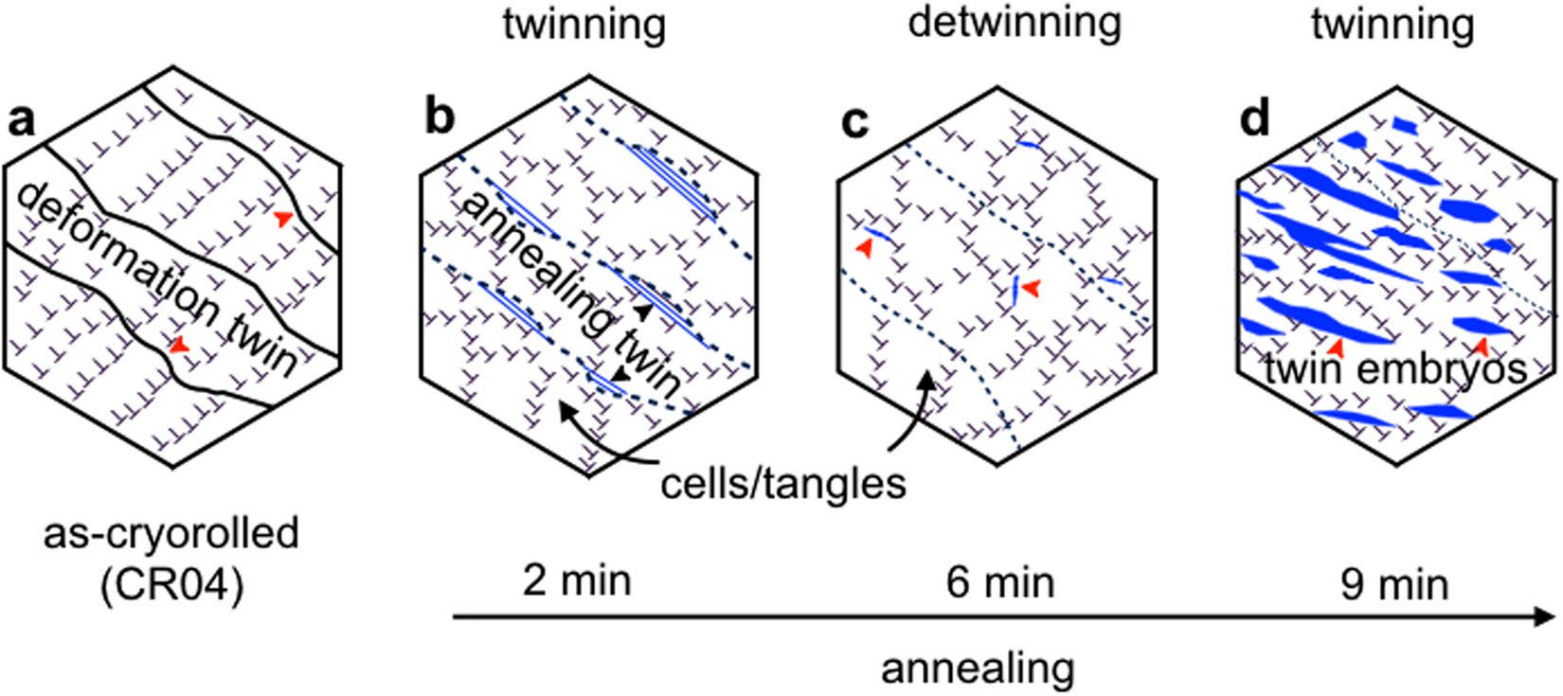
Micromechanical model showing the evolution of annealing induced nano-twins leading to anomalous hardening in fcc crystals. (**a**) Presence of deformation twins along with high density dislocations in as-cryorolled specimen (CR04). (**b**) Nucleation of nano-twins at the curved TBs and formation of low energy configuration dislocation cells/tangles after 2 min of annealing. (**c**) Dissolution of prior twins after 6 min of annealing. (**d**) Formation of new convex shape twin embryos upon further annealing (9 min).

On the basis of our experimental evidences, it is clear that the evolution of desired microstructure is the key to achieve the anomalous hardening in the fcc crystal. The two stage processing strategy (cryorolling and low temperature annealing) is schematically represented with the help of a micromechanical model, as shown in Fig. 6. Cryorolling restricts the dynamic recovery process and retains the high-density dislocation as well as the nano-twins in the microstructure (Fig. 6a). Annealing of CR04 results in the dissolution of the prior deformation twins, nucleation of new annealing twins, and rearrangement of the high-density dislocations network to form dislocation cells/tangles (Fig. 6b–d). Here, the anomalous increment in the flow stress is associated with the evolution of a highly twinned nanocrystals (Fig. 6b,d). Whereas, the nanocrystals with low value of *β* results low value (342–379 MPa) of the flow stress. We, therefore predict that evolution of such nano-twins upon controlled annealing of a microstructure with high density of structural defects (here dislocations; Fig. 2a) reduces the effective dislocation mean free path and increases the flow stress.

Our findings introduce a new strategy to strengthen fcc nanocrystals. The selection of suitable microstructure of the starting material along with the choice of proper annealing conditions are essential for the annealing induced hardening phenomena. On the basis of our results, if nano-twins and dislocations can be produced at the same scale/density in other fcc crystals, an anomalous increment in the flow stress would be achieved.

## Methods

### Cryorolling and annealing

We fabricated nanostructured α-brass (Cu-30 wt.% Zn) with varying twin density, crystallite size, and dislocation density through cryorolling up to the true strain of 1.0 (Fig. 2a). Initially, the as-cast 50 × 30 × 15 mm^3^ bars were solution treated at 560 °C for 24 h followed by air-cooling. Cryorolling experiments were performed by dipping the solution treated samples into liquid N_2_ kept at −120 °C for 30 minutes before and after each pass. The thickness reduction after each pass was 5%. The specimens were collected after achieving the true strain of 0.2 (CR02), 0.4 (CR04), 0.5 (CR05), 0.6 (CR06), 0.7 (CR07), 0.8 (CR08), 0.9 (CR09), and 1.0 (CR10). Cryorolled coupons (CR04) of 10 × 10 × 2 mm^3^ size were annealed at 165 °C (0.18T_m_) and 200 °C (0.22T_m_) up to 16 minutes in a vertical tube furnace followed by ice brine (−15 °C) quenching. The annealing temperatures are well below the stress relieving annealing (0.29T_m_ = 260 °C) and the recrystallization temperatures (0.56T_m_ = 522 °C) (Supplementary Figure S1).

### X-ray peak broadening analyses

Structural characterization was carried out using x-ray diffraction (X’pert PRO, PANanalytical) with Cu-Kα radiation. Rietveld whole x-ray profile fitting technique was adopted to estimate crystallite size, dislocation density, and twin fault probability using Material Analysis Using Diffraction (MAUD, Version 2.55) software. The software simulates a diffraction pattern by fitting a series of structural, microstructural, peak shape, width, and background parameters and compared with that of the experimentally observed pattern^28^. All the variable parameters were refined further by adopting an iterative least-squares procedure through minimization of the residual parameters^28^.

Rietveld refinement considers several analytical functions consisting of both the instrumental and structural parameters to model the experimentally measured XRD pattern. The measured XRD pattern is described by the whole profiles using a pseudo-Voigt (pV) function, whose intensity is directly related to the structure of each constituent phase. Enzo *et al*. have described the XRD profile by a convolution equation as^29^:

2 $${Y}_{c}(2\theta )=[{B}^{\ast }({I}^{\ast }A)](2\theta )+bkg$$

where, * is the convolution symbol and bkg is the four degree polynomial function reproducing the background. The symmetric part of the instrumental function *I* and the true broadening function *B*, have been represented by a pV function for both *Kα*_1_ and *Kα*_2_ peaks as follows:

3 $${\boldsymbol{p}}V(2\theta )={\sum }_{{\alpha }_{1}{\alpha }_{2}}{I}_{nt}(1-\eta ){(1+{S}^{2})}^{-1}+\eta \,exp(-\mathrm{ln}\,2\times {S}^{2})$$

where, *S* = (2*θ* − 2*θ*_0_)*/HWHM*, HWHM is the half width at half maxima of the x-ray peaks, *η* is the Gaussianity of x-ray peaks, *θ*_0_ is the Bragg angle of Kα_1_ peak, *I*_*nt*_ is the scale parameter of the pV function, and $A(2\theta )=exp[-a|2{\theta }_{m}-2{\theta }_{0}|tan2{\theta }_{0}]$ is the asymmetric part of the instrumental function. The position of Kα_1_ peak was calculated from the cell parameters considering systematic errors in the peak position. HWHM and *η* have been generated from the *D* and ${\langle {{\rm{\varepsilon }}}^{2}\rangle }^{\frac{1}{2}}$ values of the standard sample^30,31^. The *D* and the ${\langle {{\rm{\varepsilon }}}^{2}\rangle }^{\frac{1}{2}}$ in the present study were evaluated using the DELF model^28^. The *ρ*_*d*_ can be estimated from ${\langle {{\rm{\varepsilon }}}^{2}\rangle }^{\frac{1}{2}}$ and *D* according to the following equation^32^:

4 $${\rho }_{d}=\frac{2\sqrt{3}{\langle {\varepsilon }^{2}\rangle }^{\frac{1}{2}}}{Db}$$

where, Burger’s vector, *b* is considered to be 0.26 nm for Cu-30 wt.% Zn

Warren *et al*. have reported that twin faults probability (*β*), intrinsic stacking faults probability (*IF*), and extrinsic stacking faults probability (*EF*), contribute to the peak shift, anisotropic broadening, and asymmetry as^33^:

5 $${\rm{\Delta }}(2\theta )=\frac{90\sqrt{3}(IF-EF)tan\,\vartheta }{{\pi }^{2}{h}_{0}^{2}(u+{b}_{1})}\sum _{{b}_{1}}(\pm {L}_{0})$$

6 $$\frac{1}{{D}_{eff}}=\frac{1}{D}+\frac{[1.5(IF-EF)+\beta ]}{a{h}_{0}(u+b)}\sum _{{b}_{1}}({L}_{0})$$

7 $${y}_{2}-{y}_{1}=\frac{2A{b}_{1}(4.5EF+\beta )}{\sqrt{3}\pi (u+b)}\pm \frac{L}{|{L}_{0}|}\frac{1}{{c}_{2}{x}_{2}}$$

8 $${c}_{2}=1+\{\frac{\lambda }{4\pi {D}_{eff}[sin\,({\vartheta }_{0}+{x}_{2})-sin\,{\vartheta }_{0}]}\}$$

The *IF*, *EF*, and *β* probabilities are parameters in the MAUD software. Eqns (5)–(7) were used to estimate the peak shift, broadening and the asymmetry of the x-ray pattern due to *IF*, *EF*, and *β* in the FCC structure.

The MAUD software refines the background parameters, volume fractions, and 2*θ* values, etc., during the first cycle of the refinement. The structural parameters are refined during the next cycles. Here, the software matches the peak positions of both the experimented and simulated profiles. Once the peak position of both the patterns become identical, the next stage of refinement starts. The refinement of microstructural parameters starts considering the default values crystallite size (100 nm) and lattice strain (8 × 10^−4^). A reasonably good fit can be achieved by adjusting these two parameters carefully. Finally, the different fault probabilities (*IF*, *EF*, and *β*) are refined, which improves the quality of fitting. A careful handling of the refinement process yields a low weighted residual error value.

### TEM investigation

The microstructure of the cryorolled and annealed specimens were characterized using a transmission electron microscope (JEM-2100, JEOL) at an accelerating voltage of 200 kV. Thin slices were cut from the bulk sample and were mechanically polished. Finally, the specimens were dimpled followed by Ar-ion milling (PIPS-691, Gatan) with liquid N_2_ cooling facility for electron transparency.

### Compression test

Parallelepiped specimen having dimension of 2 × 2 × 4 mm^3^ were cut from the CR04 and differently annealed specimen along both the RD and TD using wire electro discharge machine. Compression tests were performed at room temperature using a universal testing machine (Tinius Olsen HSKOS) at an initial strain rate of 8 × 10^−4^/s.

## Electronic supplementary material

Supplementary Info
