# Supplementary material for: Strengthening face centered cubic crystals by annealing induced nano-twins
Source: Sci Rep. 2017 Dec 13;7:17512. doi: 10.1038/s41598-017-17848-3 (PMC5727482; doi:10.1038/s41598-017-17848-3)
Supplement: Supplementary file 1 — Supplementary Info [file 41598_2017_17848_MOESM1_ESM.doc]

**Strengthening face centered cubic crystals by annealing induced nano-twins**

Barna Roy and Jayanta Das

**SUPPLEMENTARY INFORMATION**


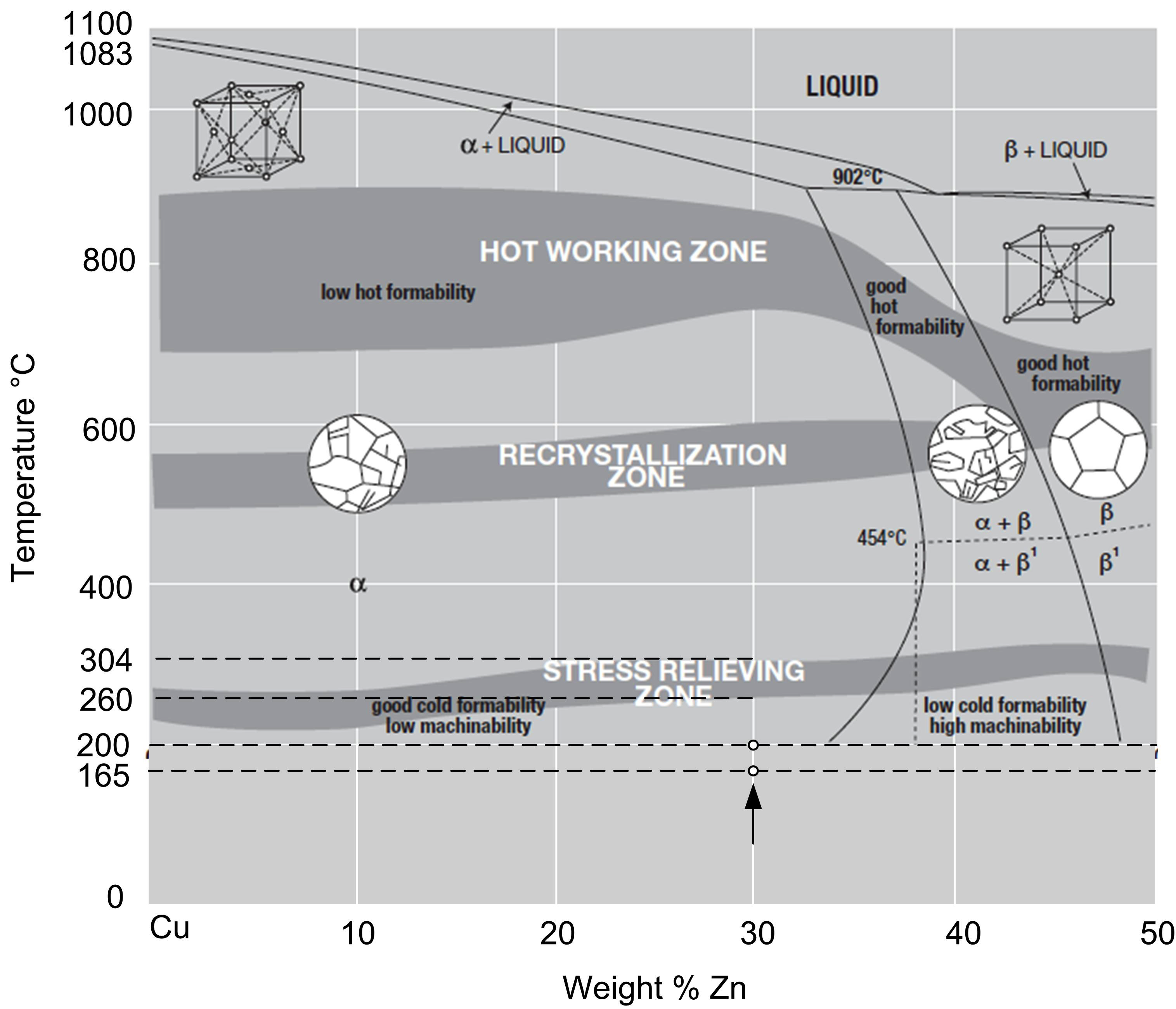


**Figure S1**| **Schematic showing different temperature range for heat treatment of α-brass.** The selected annealing temperatures in the present study for CR04 are 165 °C and 200 °C, which are well bellow the stress reliving temperature (260 °C - 304 °C) and recrystallization temperature (522 °C - 600 °C) ^1^.


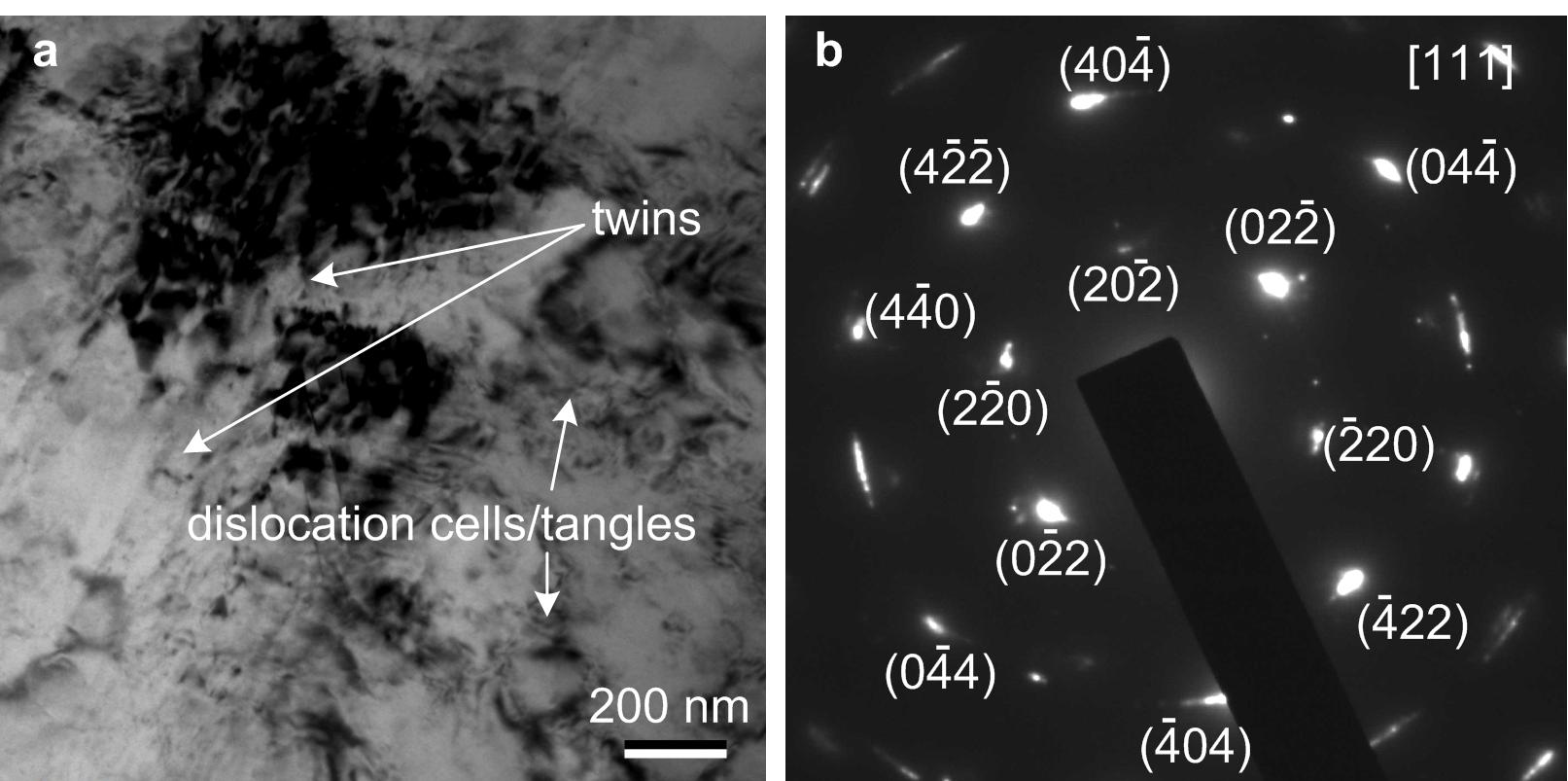


**Figure S2**| **TEM micrograph of the specimen annealed for 16 min at 165 °C.** **a,** BF microstructure showing the formation of 200-500 nm size dislocation cells/tangles with negligible amount twins. **b,** corresponding SAED pattern along [111] zone axis with weak twin diffraction spots.

Table S1. Summary of the XRD peak broadening analysis and the flow stress of the as-cryorolled (CR04) and annealed specimens

1. Callcut, V. in *The Brasses properties & applications,* (Copper Development Association, UK, 1996).

| Sample | | *D*  nm | 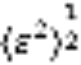  (×10^3^) | 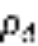  (×10^-14^) | *β* | *σ*_y_ (MPa) | |
| --- | --- | --- | --- | --- | --- | --- | --- |
| Temperature | Time (min) |  |  |  |  | RD | TD |
| CR04 | | 57 | 1.57 | 3.70 | 2.27×10^-5^ | 440±5 | 470±8 |
|  |  |  |  |  |  |  |  |
| 165 °C | 1 | 74 | 2.68 | 4.80 | 4.18×10^-5^ | 460±1 | 556±6 |
|  | 2 | 61 | 2.15 | 4.78 | 9.28×10^-4^ | 515±1 | 578±2 |
|  | 3 | 62 | 2.41 | 5.12 | 9.68×10^-5^ | 465±6 | 555±9 |
|  | 4 | 72 | 2.69 | 4.93 | 1.67×10^-4^ | 457±1 | 480±4 |
|  | 5 | 80 | 3.20 | 5.32 | 1.51×10^-5^ | 411±7 | 483±18 |
|  | 6 | 51 | 1.98 | 5.11 | 2.98×10^-6^ | 415±3 | 458±14 |
|  | 7 | 59 | 2.27 | 5.13 | 3.18×10^-7^ | 439±11 | 473±1 |
|  | 8 | 65 | 2.47 | 5.05 | 5.16×10^-5^ | 442±2 | 507±2 |
|  | 9 | 62 | 2.34 | 5.27 | 5.26×10^-4^ | 503±2 | 541±1 |
|  | 10 | 67 | 2.66 | 5.29 | 8.50×10^-7^ | 456±12 | 522±1 |
|  | 11 | 65 | 2.28 | 4.67 | 6.25×10^-7^ | 383±17 | 482±18 |
|  | 12 | 77 | 2.67 | 4.59 | 2.51×10^-6^ | 447±10 | 510±20 |
|  | 13 | 68 | 1.78 | 4.60 | 8.16×10^-6^ | 438±11 | 487±10 |
|  | 14 | 67 | 2.81 | 5.58 | 1.22×10^-6^ | 442±1 | 512±5 |
|  | 15 | 66 | 1.99 | 4.04 | 2.43×10^-5^ | 457±2 | 495±10 |
|  | 16 | 71 | 2.77 | 5.22 | 1.31×10^-6^ | 431±4 | 506±1 |
|  |  |  |  |  |  |  |  |
| 200 °C | 1 | 62 | 1.68 | 3.60 | 1.53×10^-9^ | 464±1 | 498±4 |
|  | 2 | 54 | 1.57 | 3.89 | 6.48×10^-6^ | 493±5 | 498±6 |
|  | 3 | 50 | 1.70 | 4.53 | 2.88×10^-7^ | 342±1 | 379±36 |
|  | 4 | 40 | 1.56 | 5.21 | 1.97×10^-5^ | 477±1 | 507±21 |
|  | 5 | 45 | 1.77 | 5.19 | 1.02×10^-5^ | 496±1 | 480±11 |
|  | 6 | 43 | 1.73 | 5.32 | 1.93×10^-5^ | 499±3 | 470±3 |
|  | 7 | 44 | 1.67 | 5.05 | 5.75×10^-6^ | 482±13 | 495±27 |
|  | 8 | 50 | 1.95 | 5.19 | 6.43×10^-6^ | 490±3 | 476±16 |
|  | 9 | 47 | 1.81 | 5.12 | 1.75×10^-4^ | 523±25 | 542±9 |
|  | 10 | 46 | 1.81 | 5.18 | 2.62×10^-9^ | 475±16 | 445±7 |
|  | 11 | 42 | 1.85 | 5.78 | 2.13×10^-7^ | 1446±9 | 429±2 |
|  | 12 | 64 | 1.74 | 3.62 | 2.12×10^-8^ | 498±25 | 503±1 |
|  | 13 | 51 | 1.78 | 4.58 | 3.83×10^-6^ | 492±9 | 492±38 |
|  | 14 | 40 | 1.82 | 6.06 | 9.61×10^-7^ | 491±26 | 511±1 |
|  | 15 | 46 | 2.04 | 5.81 | 1.25×10^-7^ | 489±2 | 466±6 |
|  | 16 | 55 | 1.84 | 4.44 | 3.63×10^-6^ | 479±35 | 504±8 |
